# Genome wide association study of *Escherichia coli* bloodstream infection isolates identifies genetic determinants for the portal of entry but not fatal outcome

**DOI:** 10.1371/journal.pgen.1010112

**Published:** 2022-03-24

**Authors:** Erick Denamur, Bénédicte Condamine, Marina Esposito-Farèse, Guilhem Royer, Olivier Clermont, Cédric Laouenan, Agnès Lefort, Victoire de Lastours, Marco Galardini, the COLIBAFI, SEPTICOLI groups

**Affiliations:** 1 Université de Paris, IAME, UMR 1137, INSERM, Paris, France; 2 Laboratoire de Génétique Moléculaire, Hôpital Bichat, AP-HP, Paris, France; 3 Département d’épidémiologie, biostatistiques et recherche clinique, Hôpital Bichat, AP-HP, Paris, France; 4 LABGeM, Génomique Métabolique, Genoscope, Institut François Jacob, CEA, CNRS, Université Paris-Saclay, Evry, France; 5 Département de Prévention, Diagnostic et Traitement des Infections, Hôpital Henri Mondor, Créteil, France; 6 Service de Médecine Interne, Hôpital Beaujon, AP-HP, Clichy, France; 7 Institute for Molecular Bacteriology, TWINCORE Centre for Experimental and Clinical Infection Research, a joint venture between the Hannover Medical School (MHH) and the Helmholtz Centre for Infection Research (HZI), Hannover, Germany; 8 Cluster of Excellence RESIST (EXC 2155), Hannover Medical School (MHH), Hannover, Germany; University of Warwick, UNITED KINGDOM

## Abstract

*Escherichia coli* is an important cause of bloodstream infections (BSI), which is of concern given its high mortality and increasing worldwide prevalence. Finding bacterial genetic variants that might contribute to patient death is of interest to better understand infection progression and implement diagnostic methods that specifically look for those factors. *E*. *coli* samples isolated from patients with BSI are an ideal dataset to systematically search for those variants, as long as the influence of host factors such as comorbidities are taken into account. Here we performed a genome-wide association study (GWAS) using data from 912 patients with *E*. *coli* BSI from hospitals in Paris, France. We looked for associations between bacterial genetic variants and three patient outcomes (death at 28 days, septic shock and admission to intensive care unit), as well as two portals of entry (urinary and digestive tract), using various clinical variables from each patient to account for host factors. We did not find any association between genetic variants and patient outcomes, potentially confirming the strong influence of host factors in influencing the course of BSI; we however found a strong association between the *papGII* operon and entrance of *E*. *coli* through the urinary tract, which demonstrates the power of bacterial GWAS when applied to actual clinical data. Despite the lack of associations between *E*. *coli* genetic variants and patient outcomes, we estimate that increasing the sample size by one order of magnitude could lead to the discovery of some putative causal variants. Given the wide adoption of bacterial genome sequencing of clinical isolates, such sample sizes may be soon available.

## Author summary

*Escherichia coli* is a human gut commensal but also a main opportunistic pathogen responsible for extra-intestinal infections such as bloodstream infections. These infections are particularly severe and have been increasing in frequency. Furthermore, *E*. *coli* has become more resistant to antibiotics, rendering treatment difficult. To decipher whether bacterial genetic traits are linked to infection establishment and outcomes, we carried out a genome-wide association study for a collection of almost one thousand of *E*. *coli* bloodstream infection isolates from the Paris area, France. We did not find genetic variants linked to infection severity, but we identified a gene cluster (*papGII*) associated with the establishment of bloodstream infection through the urinary tract. Our simulations indicate that the failure to identify variants linked to infection severity could be due to an insufficient sample size, which could be easily solved in the future as pathogen genome sequencing becomes common in clinical practice.

## Introduction

*Escherichia coli* bloodstream infections (BSI) represent an increasing public health burden as (i) they exhibit high mortality (between 10 and 30%) [1,2], (ii) its worldwide prevalence is increasing since the 2000s [3], and (iii) antimicrobial resistance is rising in *E*. *coli* [3], which could impact patients’ management and infection outcome. Molecular epidemiology of BSI has been refined in the last few years thanks to whole genome sequencing. *E*. *coli* has a clonal population structure [4] with the delineation of at least eight phylogroups (A, B1, B2, C, D, E, F and G) [5]. Strains responsible for BSI belong mainly to a few clonal lineages including sequence types (ST) ST131, ST73, ST95, ST69, and all of the B2 and D phylogroups [5]. Until now, classical multivariate analyses have identified host factors and portal of entry as the major determinants of a patient’s death, while bacterial genetic traits have been associated with a smaller effect size to mortality or only in a subset of studies [1,2,6–10].

Bacterial genome wide association studies (GWAS) are now common thanks to an increase in sequencing capacity and specific computational tools [11]; in *E*. *coli* they have allowed the identification of genetic traits linked to pathogenicity in avian strains [12], invasiveness in urinary tract infection (UTI) strains [13], and isolation source [14]. However, they failed to identify genetic markers of disease severity in Shigellosis [15]. This could have multiple explanations including small sample sizes that can lead to insufficient power to find causal variants. Disease severity (e.g. patient death) is a trait that is not under selection as it doesn’t provide a reproductive advantage, and is therefore less likely to evolve independently across multiple lineages, which in turn makes it less likely to be found through bacterial GWAS [16]. Furthermore, as opposed to antimicrobial resistance which is often caused by a handful of genetic variants, disease severity might involve multiple genetic loci, each with small effects, which are harder to discover.

Identifying microbial genetic elements that contribute to the outcome of BSI is of interest to i) better understand the molecular mechanisms of microbial infection, and ii) improve patient care and prediction of clinically-relevant bacterial traits based on microbial genomics data, which is increasingly becoming available with very low turnaround time [17]. In this context, we performed GWAS on data from two large clinical observational prospective multicentric studies from the Paris area (Septicoli [10] and Colibafi [8]) involving a total of 912 adult patients with *E*. *coli* BSI. We used the clinical information from each patient, such as age, comorbidities and treatment as covariates to reduce the influence of host factors in the association analysis [18]. We then performed a power analysis using simulated genotypes and phenotypes to understand which sample size would be appropriate to reach an ever higher statistical power. As microbial whole genome sequencing costs keep reducing we argue that a 10-fold increase in sample size for this kind of studies is likely to be available in the near future.

## Results

### A combined dataset of 912 BSI patients with matching clinical data and bacterial isolates whole genomes

In this study we combined data from two similar clinical studies (Colibafi [8] and Septicoli [10]), conducted across 11 teaching hospitals, belonging to the same institution, the “Assistance Publique-Hôpitaux de Paris” (AP-HP), across and around Paris, France. The earlier study (Colibafi, 2005) originally included 1,051 patients across the whole of France, with information about bacterial genetic determinants obtained through PCR molecular assays; in this study we kept only those 365 samples originating from 8 hospitals from the Paris area to avoid geographical biases, due for instance to the specialization of some hospitals in the treatment of specific pathologies. From the later study (Septicoli, 2016–7) we kept all the 545 samples from 7 hospitals in the Paris area. Bacterial genomes of these samples from both studies, generated by Illumina technology, were available [19].

We focused on three outcomes for the patients represented in the combined dataset, namely death at 28 days, presence of a septic shock and admission to an intensive care unit (ICU); we note that these outcomes are not mutually exclusive. The prevalence of these outcomes in the two studies was 10.7%, 24% and 14.6%, for death, septic shock and admission to ICU, respectively (S1 Fig). The prevalence of death and admission to ICU was very similar between the two studies, with 12.5% and 9.5% of deaths in the Colibafi and Septicoli studies, respectively, and admission to the ICU reported for 12.5% and 16% of patients. On the other hand, we found a much higher incidence of patients experiencing septic shock in the Septicoli cohort as opposed to Colibafi: 32.5% of patients versus 11.4%. These variations may be due to the different hospitals contributing the clinical data between the two studies: indeed, even though both studies are exclusively focused on the AP-HP teaching hospitals in Paris, only 4 out of 11 hospitals are included in both studies [19]. We additionally focused on the reported portal of entry of the BSI, which has been previously found to be predictive of patient outcome; the urinary and digestive tract portals of entry were the most prevalent in the combined dataset—58.2% and 35.6% of patients, respectively. The other reported portals of entry all had a prevalence below 5%, and we therefore chose to only use the urinary and digestive tract portals of entry for all subsequent analyses. We found that entry through the digestive tract was reported for 41.8% of the patients in the Septicoli study, compared to 26.6% in the Colibafi study, which again may be due to differences in the hospitals providing the data for both studies. The age distribution between the two studies is comparable, with median age of the patients being 67 and 69 years in the Colibafi and Septicoli studies, respectively (S1 Fig). To reduce the influence of these differences between the two studies on our analyses, we introduced the study provenance as a covariant in the combined dataset (S1 Table).

### The pathogen portal of entry is associated with BSI outcomes

We found that several clinical variables are associated with the three patient outcomes, consistent with earlier analyses on the two studies alone [8,10] (Table 1). Among other variables, entry through the pulmonary and digestive tract were associated with death (odds ratio 2.88 and 1.51 and p-values 8E^-5^ and 0.006, respectively), while entry through the urinary tract was found to be negatively associated (odds ratio 0.51, p-value 2E^-5^). Entry through the pulmonary tract was also associated with septic shock (odds ratio 2.12, p-value 4E^-3^), while entry through the digestive tract was associated with patients being admitted to the ICU, among other variables (odds ratio 1.53, p-value 1E^-3^). When combining all clinical variables with association p-value < 0.1 into a multivariate analysis (Table 2, see Materials and methods) we found that portal of entry was again the dominant variable associated with patient outcomes, together with study provenance. In particular entry through the pulmonary tract was significantly associated with a patient’s death (odds ratio 2.40, p-value 0.003), while entry through the urinary tract was negatively associated (odds ratio 0.64, p-value 0.008). Entry through the pulmonary tract was also significantly associated with a patient experiencing a septic shock (odds ratio 2.10, p-value 0.005), while entry through the digestive tract was associated with a patient being admitted to the ICU (odds ratio 1.57, p-value 0.002). This analysis underscores the influence of the *E*. *coli* portal of entry on BSI outcomes; if the portal of entry was influenced by a bacterial genetic variant, we could expect to find it through a statistical association.

**Table 1:** Univariate analysis on the combined dataset. Only clinical variables significantly associated with BSI outcomes are shown. CI, confidence interval.

Patient outcome	Clinical variable	Odds-ratio [95% CI]	P-value
death	urinary tract	0.51 [0.38–0.69]	2E^-5^
	pulmonary tract	2.88 [1.70–4.87]	8E^-5^
	malignant tumor	1.75 [1.30–2.35]	2E^-4^
	digestive tract	1.51 [1.12–2.04]	0.006
	chronic alcoholism	1.74 [1.16–2.60]	0.007
	immunosuppression	1.50 [1.11–2.02]	0.007
	active smoking	1.56 [1.11–2.19]	0.01
septic shock	pulmonary tract	2.12 [1.27–3.55]	0.003
admission to ICU	cirrhosis	1.99 [1.37–2.89]	3E^-4^
	digestive tract	1.53 [1.18–1.99]	0.001
	active smoking	1.59 [1.17–2.16]	0.003

**Table 2:** Multivariate analysis on the combined dataset. Clinical variables with p-value < 0.01 are reported for each patient outcome; the intercept is excluded. CI, confidence interval.

Patient outcome	Clinical variable	Odds-ratio [95% CI]	P-value
death	study: septicoli	0.59 [0.41–0.83]	0.003
	pulmonary tract	2.40 [1.33–4.20]	0.003
	urinary tract	0.64 [0.45–0.89]	0.008
septic shock	study: septicoli	2.54 [1.96–3.33]	5E^-12^
	pulmonary tract	2.10 [1.25–3.51]	0.005
admission to ICU	digestive tract	1.57 [1.19–2.08]	0.002

### The pathogen phylogroup is associated with the portal of entry but not with BSI outcomes

**Fig 1:**
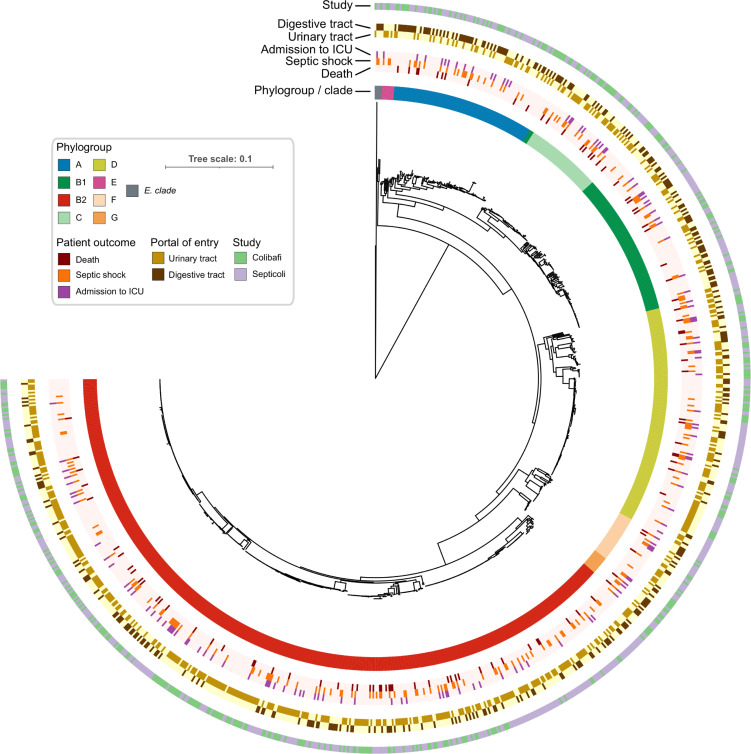
Core genome phylogenetic tree of the 912 *E*. *coli* isolates used in this study. Each ring reports the main bacterial and clinical variables of this study. The light color in the rings related to patient outcomes and portals of entry indicates the absence of the trait.

We found that no *E*. *coli* phylogroup was associated with patient death (p-value > 0.01), consistent with earlier analyses from the two separate studies [8,10], in contrast to what we previously observed in a mouse model of BSI, in which we found that the B2 phylogroup was associated with the death of the animal [20]. We also observed no association between an isolate’s phylogroup and a septic shock or admission to the ICU. The absence of these genetic background effects does not imply that there are no “locus effects”, meaning that individual genetic variants may still be found to be associated with patients’ outcomes. On the other hand, we found a strong association between the isolates’ phylogroup and the urinary and digestive tract portals of entry; phylogroup B2 was associated with the urinary tract, while phylogroups A and, B1 were associated with the digestive tract (p-value < 0.01, S2 Table). Such similarity between the two phenotypes is not surprising given the low prevalence of the other portals of entry, leading to two almost mutually exclusive traits (Fig 1).

### Bacterial genetic factors can explain a significant fraction of the variation of the route of infection

**Fig 2:**
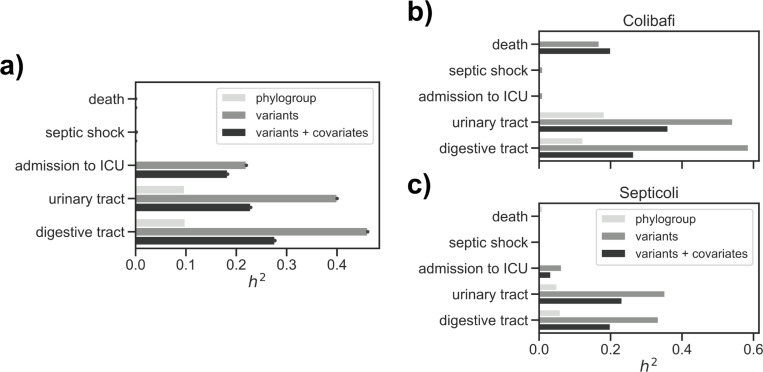
Narrow-sense heritability (*h*^*2*^) estimation for the target variables on the combined dataset. a) Heritability estimates in the two studies combined, using a covariance matrix generated from the isolates’ phylogroup (phylogroup), a kinship matrix generated from the unitigs presence/absence matrix (variants), and the same kinship matrix conditioned with the clinical variables (variants + covariates). b) Heritability estimates in the Colibafi and c) Septicoli cohorts alone.

We used narrow-sense heritability—the fraction of phenotypic variance that is explained by additive genetic effects [21]—to estimate whether we could expect to find bacterial genetic variants in association with the three patient outcomes or the two main portals of entry. Since we found that clinical variables and the pathogen’s phylogroup are associated with our target variables, we measured heritability in three ways: using the phylogroup alone as a genetic effect [16], and using a kinship matrix generated from the whole genetic variation as encoded by unitigs, alone or conditioning the analysis with the clinical variables in order to account for confounding factors (Fig 2). We found that phylogroups could explain 10% of the variation for both the urinary and digestive tract infections (95% CI 0.01% - 48.3% for both), but none for any of the three patient outcomes. Overall genetic variation could however explain 22% (95% CI 0% - 95.3%) of the variation in admission to the ICU, which was negligibly reduced to 18% (95% CI 0% - 96.4%) when considering clinical covariates. While this may seem to indicate that the pathogen genetic variation might influence whether a patient will eventually need intensive care, we noted that this relatively high heritability was present in the Septicoli cohort alone (Fig 2B and 2C). We didn’t however find such a discrepancy between the two studies when we estimated the heritability for the portals of entry using either overall genetic variation alone or after conditioning. This indicates that there may be confounding factors that contribute to the decision to change a patient’s treatment which vary between the two studies. This is unsurprising, as the decision to admit a patient to the ICU can depend on the subjective assessment of a physician considering a patient’s comorbidities, as well as other subtle differences in care protocols.

Conversely, the estimated heritability due to genetic effects for the portals of entry varies in magnitude between the combined dataset and the two cohorts alone, but we nonetheless found it to be > 0 in the three datasets. In particular, we found that genetic effects could explain 40% and 46% of the variance of urinary and digestive tract portals of entry, respectively (95% CI 0% - 95.7% and 0% - 95.8%, respectively), which is more than four times the variance explained by the isolates’ phylogroup (Fig 2A). This suggests that a genome-wide association analysis is likely to discover genetic variants associated with the portal of entry for BSI. This relatively high fraction of the phenotypic variability explained by genetic effects is however reduced when conditioning it on other clinical variables (23% and 28% for the urinary and digestive tract portal of entry, respectively, 95% CI 0% - 96.4% for both traits), which again underscores the influence of host characteristics in determining the establishment of bloodstream infections.

### The *papGII* operon is associated with the pathogen’s entry through the urinary tract

In order to account for both core and accessory genome genetic variability, which is one of the main differences between GWAS studies in human and bacterial datasets, we associated unitigs generated from a de Bruijn graph of all the bacterial isolates against the target variables [22,23]; namely the three patient outcomes and the two major portal of entry for BSI. We used a linear mixed model for the association, which has been shown to better correct for the influence of bacterial population structure in the association [24]. In order to account for the host and clinical factors on target variables, we conducted the association with the clinical variables as covariates [25]. Since our earlier analysis indicated that the portal of entry can influence patient outcome, we added this information as covariates when looking for bacterial genetic factors associated with the three patient outcomes.

**Fig 3:**
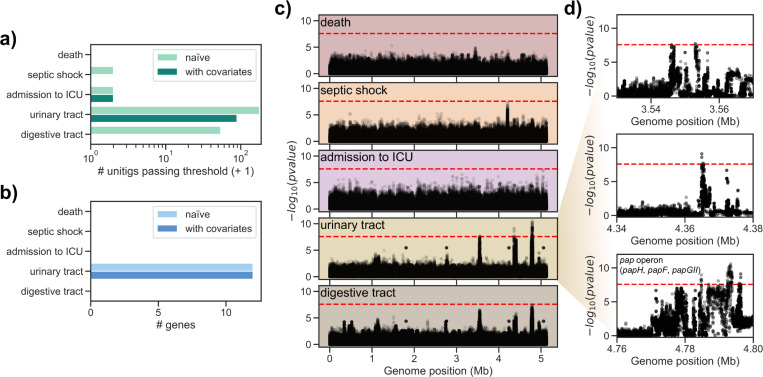
Genome-wide association analysis results on the combined dataset. a) Number of unitigs passing the multiple testing correction p-value threshold for each target phenotype. b) Number of genes with significantly associated unitigs mapped to them for each target phenotype. c) Manhattan plots for tested unitigs mapping to *E*. *coli* IAI39 for each target phenotype; red dashed line indicates the p-value threshold used to call significant associations. d) Zoomed-in Manhattan plots for the urinary tract trait. Genes annotated with a gene name in *E*. *coli* IAI39 and with associated unitigs are indicated in the third subpanel.

Consistent with the heritability estimates, we found few or no unitigs associated after multiple testing correction with either the death of the patient (none for both the naïve and conditioned association), the presence of septic shock (one for the naïve association and none for the conditioned association) and admission to ICU (one for both the naïve and conditioned association). Conversely, we found a larger number of unitigs to be associated with either portal of entry; 177 and 53 for the urinary and digestive tract, respectively, when running a naïve association, and a lower number when adding clinical covariates, with 88 unitigs passing the significance threshold for the urinary tract, and none for the digestive tract, respectively (Figs 3A, S2, and S3 Table). Finding an association between individual unitigs and a phenotype of interest may be due to chance, even after multiple testing correction and the inclusion of covariates [26]. To reduce the influence of these factors on the results of the associations, we conducted a stringent analysis when mapping the unitigs back to each bacterial isolate; briefly, we took steps to exclude those unitigs that are mapped to multiple genes across all strains or that are found in a low number of strains (see Materials and methods). After this stringent mapping step, we found no genes with associated unitigs mapped to them for the three patient outcomes and entry through the digestive tract, and 12 genes for the urinary tract portal of entry, independently on whether we used the clinical covariates in the unitig association step (Fig 3B, S4 Table and S1 Data). The absence of any associated gene with the three patients’ outcomes is in agreement with the heritability estimates, and with our argument that the relatively high heritability for the admission to the ICU may be the result of confounders.

We found similar effect sizes reported for all tested unitigs for the two portals of entry (Pearson’s r for the odds-ratio -0.76), but with opposite signs, which is likely the result of the two traits being almost exactly mutually exclusive (S3 Fig). This similarity in the association results is also evident from the Manhattan plot of the two traits (Fig 3C), which shows peaks in the same region of *E*. *coli* IAI39 chromosome.

Among these 12 genes associated with urinary tract, six belonged to the *pap* operon or in its immediate vicinity; the genes in this operon encode for a type P pilus, which has been shown to interact with glycolipids present on uroepithelial cells and is therefore believed to be one of the main defining loci for severe UTI. We found that the *papGII* variant of the *papG* gene encoding for the adhesin part of the tip was associated with entry through the urinary tract (S4 Fig). The PapGII adhesin is mainly found in acute pyelonephritis and binds preferentially to Gb4 (GalNAcβ1-3Galα1-4Galβ1-4GlcCer), which is abundant in the upper urinary tract of humans [27]. We found another three genes associated with both portals of entry and encoded in the vicinity of the *pap* operon, all with high sequence similarity (blastp sequence identity > 95%) to genes annotated as phosphoethanolamine transferases, or *opgE*. This gene is involved in the biosynthesis of osmoregulated periplasmic glucans (OPGs), which in turn regulate motility and secretion of exopolysaccharides and are considered virulence factors for Gram-negative species [28–31]. We found these putative *opgE* genes encoded in the vicinity of phage-derived integrase genes (annotated as *intA* and *intS*). The putative *opgE* gene was encoded in the near vicinity of the *pap* operon (distance < 15kbp) in 118 strains, and an even shorter distance (< 10kbp) between the *pap* operon and the edge of its contig for those strains (201) in which the *pap* operon and the putative *opgE* gene were encoded in separate contigs (S3 Fig). We therefore concluded that both the putative *opgE* gene and the integrase genes are part of the same genetic island that may have been acquired through horizontal gene transfer across *E*. *coli* strains [13].

Since we observed a strong association between the portal of entry and phylogroup B2, we also ran an association that included only B2 strains and conditioned with the clinical variables (N = 492). Similarly to the analysis with the full dataset, we found one unitig associated with admission to ICU, 46 with entry through the urinary tract, and eight with entry through the digestive tract (S5 Fig and S3 Table), which we mapped to one, three and four genes, respectively (S4 Table). For both portals of entry we found *papGII* to be associated, as well as *papH* and *papD* for the urinary tract and digestive tract, respectively; as observed for the full dataset, the association sign is positive for the urinary tract and negative for the digestive tract. A gene annotated as *mdoB* was negatively associated with admission to ICU in this lineage specific analysis; this gene is also involved in the biosynthesis of osmoregulated periplasmic glucans (OPGs).

### A larger sample size could reveal additional bacterial factors involved in BSI

**Fig 4:**
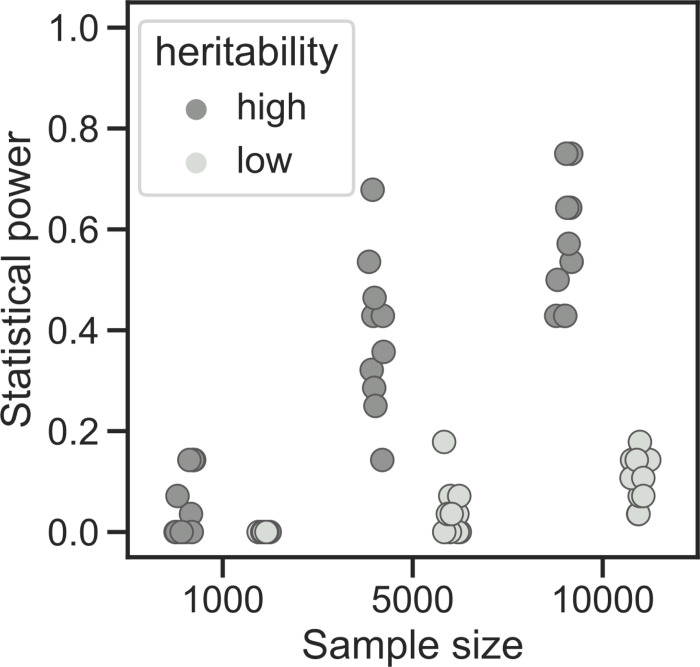
Power simulations. The proportion of causal variants passing the significance threshold is reported for each sample size and heritability for the simulated phenotypes.

Our heritability estimates and association results are in good agreement both with previous results about the difficulty of finding bacterial genetic elements associated with virulence from clinical cohorts [16] and with the importance of the *pap* operon in enabling severe UTI [13]. We next asked whether it would theoretically be possible to find even more associations from cohorts measuring *E*. *coli* BSI; would an increase in sample size lead to the discovery of more bacterial genetic factors able to affect the establishment and the outcome of BSI? To answer this question, we generated a dataset of 10,000 simulated genomes—one order of magnitude higher than the dataset presented in this study—with mutation and recombination rates similar to those of *E*. *coli*, and two phenotypes with either “high” or “low” heritability (0.2 and 0.05, respectively) [26]. For each phenotype we selected 28 causal variants with a range of effect sizes. We then ran a GWAS on the full dataset and in two smaller samples, in order to determine the empirical statistical power (Fig 4). In this simulated dataset an increase in sample size by an order of magnitude would be needed to discover most of the causal variants (mean recall 57%) for the phenotype with high heritability, which is a large increase from the sample size most similar to this study (1,000 samples, mean recall 5%). Conversely, we found that for the low heritability scenario only a relatively low statistical power (mean recall 10%) could be achieved with a large sample size of 10,000 samples, and no power when using 1,000 samples (mean recall 0). While this simulation cannot be directly compared with the genetics of complex bacterial phenotypes such as BSI caused by *E*. *coli*, it points to the theoretical possibility of further refining these results if a larger set of samples could be assembled. This could prove particularly fruitful if patient outcomes are indeed influenced at least partially by bacterial genetic factors.

## Discussion

In this study we leveraged the clinical and genetic data of two very similar BSI clinical cohorts in order to test whether *E*. *coli*’s genetic variation has an influence on the course of severe bloodstream infections. As opposed to our previous analysis using a well-controlled mouse model of sepsis where GWAS identified iron capture systems as main drivers of virulence [20], we did not find a clear locus effect for the three patient outcomes tested here. This is in agreement with a previous work in which we used 60 *E*. *coli* strains derived from bacteraemic patients and tested their virulence in the mouse model, looking for genetic determinants for the clinical severity of infection. Indeed, virulence based on an animal model was correlated with bacterial virulence determinants but not with pejorative clinical outcome of BSI [32]. In fact, the animal model is a controlled environment, as the individual tested are healthy and homogeneous (same sex, age, weight and diet) and the standardized inoculation uniformizes the portal of entry, thus allowing for an unbiased evaluation of the intrinsic virulence of each strain [33]. But an important limitation of the mouse sepsis model is that subcutaneous injection in the mouse neck does not reflect the pathophysiological processes of the portal of entry, thus skipping the first steps of BSI. The data presented here once more seems to point to either a negligible influence of bacterial genetic variation on infection outcomes when compared to host and clinical factors, a complex trait influenced by multiple loci, or to a lack of statistical power due to a relatively low sample size.

The results from the heritability analysis from this dataset of combined cohorts is mixed in this regard, as we found that the variance in a patient’s death or septic shock is not explained by bacterial genetics, while we found that locus effects may explain up to 22% of the variance in admission to ICU. When we broke down this analysis in the two cohorts alone, we observed that this relatively high heritability is only observed in the Septicoli cohort. As the decision to change the care of a patient is a complex decision dependent on the subjective assessment of clinicians and other hospital-specific policies, we believe that this high estimate may be the result of confounders. A more objective measure of disease burden may therefore be needed in order to properly test for the influence of bacterial genetics on BSI outcomes, together with an increase in sample size, as suggested by our simulations.

On the other hand, we found a clear association between the *pap* operon and surrounding genes and the urinary route of entry for BSI. This agrees with an earlier study with a similar sample size that looked specifically at invasive UTI [13]. We can point to a common theme in the genome-wide association studies so far conducted in *E*. *coli* infection models: the main associated genetic elements come from fairly frequent (~50% of the population) pathogenicity islands that have been previously described, sometimes decades before the ubiquity of genomic data made GWAS studies feasible [34–39]. One can then wonder whether these approaches are likely to ever lead to the discovery of previously undescribed genetic variants able to modulate the establishment of disease and its outcomes. We argue that as genomic sequencing of pathogens is becoming a routine part of clinical or epidemiological practice [40–42], we will likely eventually reach very large sample sizes, similar to what is currently available for human GWAS studies [43], and possibly larger, as has been recently shown for SARS-Cov-2 genomic epidemiology efforts [44,45]. Apart from increasing the power to discover genetic variants associated with a phenotype, a large sample size would allow for the discovery of rare or ultra-rare variants, which in turn may have a relatively large influence on the phenotype of interest, alone or collectively [23], as has recently been appreciated in the study of human traits and disease [46]. In particular, pooling rare variants with similar effects through burden testing approaches might uncover associations with a relatively common trait such as virulence. Additionally, focusing the analysis on a specific phylogroup known to be associated with a trait might help to boost statistical power, as we have shown when using only isolates belonging to the B2 phylogroup.

In the context of bacterial infection, in which we and others have shown how host factors contribute to a large extent, a further help will likely come from including the host genetic variation into the association. A joint human/bacterial association analysis may however require an even larger sample size in order to account for potential interactions between host and bacterial genetic elements [16]. Taken together, the assumed inevitability of clinical genome sequencing together with careful recording of host and clinical data may eventually lead to comprehensively cataloging the fraction of *E*. *coli* genetic variants that influence bloodstream infections.

## Materials and methods

### Ethics statement

Both multicenter clinical trials were approved by ethic committees. The Colibafi study was approved by the French Comité de Protection des Personnes of Hôpital Saint-Louis, Paris, France (approval #2004–06, June 2004). The Septicoli study was approved by the French Comité de Protection des Personnes Ile de France n°IV (IRB 00003835, March 2016) and was registered on clinical trials in September 2016 (ClinicalTrials.gov Identifier: NCT02890901). Because of their non-interventional nature, only an oral consent from patients was requested under French law. Both studies conformed to the principles of the Helsinki declaration.

### Dataset

The Colibafi and Septicoli studies were prospective observational cohort studies conducted in tertiary-care teaching hospitals in the Paris area. The Colibafi study was performed in 8 hospitals representing a total of 3,900 adult acute care beds whereas 7 hospitals were included in the Septicoli study, accounting for 5,800 acute care beds. Four hospitals were common between the two studies (i.e. 2,900 acute care beds). All the hospitals belong to the same institution, the “Assistance Publique-Hôpitaux de Paris” network, which accounts for a total of 13,000 adult acute care beds with a homogenous management for most bacterial infections. These hospitals receive about 10 million patients each year. As the Paris area is home to 12 million people (18% of the French population), our study can be considered as representative of the French capital, characterized by a high density of inhabitants and multinational exchanges. Adult patients with *E*. *coli* BSI were included; patients receiving vasopressors before the onset of BSI were excluded, as were patients that experienced subsequent BSI episodes during each study’s period (i.e. only samples and data from the first episode were included). *E*. *coli* BSI was defined as the isolation of *E*. *coli* from at least 1 blood culture bottle. Sepsis and septic shock were defined as recommended by the 2016 Third International Consensus Definitions for Sepsis and Septic Shock (Sepsis-3) [47]. Data were prospectively collected by clinicians in each centre on two separate visits: Visit 1 corresponded to the time of BSI (the day the blood culture was drawn; data were collected retrospectively 24-48h hours later, once the blood culture had grown) and Visit 2 corresponded to the day of discharge or in-hospital death (or day 28 if the patient was still hospitalized). For each episode, the first *E*. *coli* strain collected in the blood culture was identified. The primary endpoint was vital status at discharge or day 28 (i.e. Visit 2). The likely portal of entry was established according to clinical and/or radiological characteristics of the episodes and the isolation of *E*. *coli* from the presumed source of infection. When *E*. *coli* could be isolated from the source of infection, the portal of entry was assigned on the basis of firm clinical suspicion. In each centre, an infectious diseases clinician and a microbiologist were in charge of including patients and completing the case report form (see Colibafi and Septicoli groups in the Acknowledgments section). A steering committee was in charge of implementation and a scientific committee responsible for scientific overview.

From the combined dataset we removed those variables with more than 15% of missing values (whether the patient had received a transplant, neutropenia, pregnancy status, body mass index, patient discharge route), and we added a binary variable to record the study provenance of each sample. We imputed the remaining missing values using the MICE package, v3.12.0, using 15 iterations [48]. The raw and imputed combined datasets’ summary statistics are available as S1 Table.

### Univariate and multivariate analysis

We tested the association between clinical variables and patient outcome in a similar way as it was done in the original studies [8,10]^.^ Briefly, we first applied a min-max scaler to the age variable to bring it in the [0–1] range. For each patient outcome we then tested each clinical variable using a logistic regression as implemented in the statsmodels package, v0.11.1, using the study provenance as a covariate. We used those variables with association p-value < 0.1 to run a multivariate logistic regression, using a backward stepwise selection method to construct the final model, using the MASS package v7.3_51.3 [49].

### Whole genome sequencing and annotation

Bacterial genomes were sequenced using Illumina NextSeq technology as previously described [19]. The genomes from the Colibafi and Septicoli collections are available (Bioproject PRJEB39260 and PRJEB35745, respectively). All genomes were assembled with shovill version 1.0.4 using SPAdes v3.13.1 [50] and standard parameters, and then annotated with Prokka 1.14.5 [51]. A phylogenetic tree was computed from a core genome multiple sequence alignment, as computed by Roary v3.12 [52], using IQ-TREE v1.6.12 [53], under the GTR+F+I+G4 model. The tree was visualized using the iTOL web interface [54]. We collapsed all genes encoded in the sequenced genomes into gene families using panaroo v1.2.4 [55] with default parameters.

### Heritability estimates

We estimated narrow-sense heritability for the five target variables, using two different covariance matrices; one built from the phylogroup membership of each strain and another using a kinship matrix built from the unitigs presence and absence matrix derived from the input genomes (see next section). We excluded unitigs present in all samples and sampled 5% of the remaining unitigs. For the latter covariance matrix we also used the same clinical covariates as in the GWAS analysis (see below). We used Limix v3.04 [56], assuming normal errors for the point estimate and we computed the 95% confidence intervals using the ALBI package (commit 90d819e) [57].

### Association analyses

We derived unitigs by constructing a compressed de Bruijn graph from the input genomes, using unitig-counter v1.1.0 [22,23]. We computed the distance between each pair of samples by using mash 2.2.2 [58] with a sketch size of 10,000; we used the resulting distance square matrix to compute associations between phylogroups and each target variable, using pyseer v1.3.6 [59]. We tested for locus effects using the unitigs presence/absence vector with the FastLMM [60] linear mixed-model and a kinship matrix derived from the unitigs presence and absence matrix, as described in the previous section, using pyseer v1.3.6 [59]. We run two associations; a “naïve” one that accounted for population structure only, and one additionally conditioning on the clinical variables (“with covariates”). For the three patient outcomes we used all available variables as covariates with the exception of “death”, “septic shock” and “admission to ICU”, but including the portals of entry, which were excluded when those were the target variables. All the clinical variables used as covariates are described in S1 Table. We determined a significance threshold by counting the number of unique unitigs presence/absence patterns tested, which reduces the risk of excessively deflating association p-values. We mapped the unitigs passing the significance threshold back to all input genomes and their genes using bwa v0.7.17-r1188 [61] and bedtools v2.30.0 [62,63], using the output of panaroo to assign each unitig to a gene cluster. The unitigs were further filtered to reduce the number of spurious associations: unitigs were excluded if they were shorter than 30bp, if they were mapped to multiple locations in each genome, if they mapped to less than 9 samples (~1% of the total sample size) and if they were mapped to more than 10 different genes across all samples. We further annotated the gene families with mapped unitigs by taking a representative protein sequence from all genomes encoding each gene and using it as an input for eggnog-mapper v2.1.3 [64]. The same approach was used to run associations for isolates belonging to the B2 phylogropup.

We tested for the association of rare variants (minimum allele frequency < 1%) by performing a burden test, that is, we performed associations between deleterious rare variants in each gene separately and the five target phenotypes. We derived short variants from each sample against the complete genome of *Escherichia coli* IAI39—which belongs to phylogroup F—using snippy v4.6.0 and annotated them using SnpEff v5.0 [65]. We then merged the individual VCF files and filtered for rare variants using bcftools v1.13 [66]. We further filtered the resulting variants according to their annotation: variants annotated as “disruptive”, “frameshift”, “start codon loss”, “stop codon gain”, and “stop codon loss”; for missense variants we assessed the likelihood that they were deleterious to protein function using the SIFT algorithm, as implemented in the SIFT4G package v2.0.0 [67], using the uniref50 subset of the Uniprot database [68] (downloaded on June 16, 2021) to construct the multiple sequence alignments. We considered a missense variant to be deleterious if the protein residue had a median information content below 3.25 and score < 0.05. The association was run in a similar way as the one with common unitigs (linear mixed model and clinical covariates) using pyseer v1.3.6 [59]. No significant hit was found with this association method.

### Power simulations

We performed a statistical power analysis to test whether an increase in sample size could lead to the discovery of additional variants associated with a binary phenotype with heritability similar to that estimated in this study. We used the BacGWASim package v2.1.1 [26] to generate both simulated variants and phenotypes. We simulated 10,000 bacterial genomes each 1,000,000 bp long, using a mutation rate of 0.06 and recombination rate of 0.01. We then simulated two binary phenotypes: one with a “high” (0.2) and one with a “low” (0.05) heritability; for both phenotypes we assumed a prevalence of 50% and generated 10 sets of 28 causal variants with minimum allele frequency of 10%. For each batch of simulated phenotypes we ran an association with pyseer v1.3.6 [59] using logistic regression and population structure correction using the first four components of the multidimensional scaling obtained from the samples pairwise distance matrix computed using mash v2.2.2 [58]. Statistical power was computed as the proportion of causal variants that passed the significance threshold, computed by counting the number of unique presence/absence patterns for all tested variants.

### Computer code

Apart from the software packages mentioned in the previous sections, the following were used to run the analyses and generate the visualizations presented in this work: pandas v1.2.2 [69], numpy v1.20.0 [70], scipy v1.6.0 [71], matplotlib v3.3.4 [72], seaborn v0.11.1 [73], biopython v1.79 [74], reportlab v3.5.68 [75], gffutils v0.10.1, jupyterlab v3.0.7 [76]. Most of the analysis were incorporated in a reproducible pipeline using snakemake v6.5.0 [77] and conda v4.10.3 [78,79].

## Supporting information

S1 Fig Clinical variables of the combined dataset (912 BSI samples). a) Proportion of the three patient outcomes after BSI and their portals of entry. b) Scatterplot of the proportion of all binary clinical variables in the two studies, highlighting the major differences. c) Violin plot showing the patients’ age distribution between the two studies.
(EPS)

S2 Fig Core genome phylogenetic tree and presence/absence matrix for the 88 unitigs significantly associated with entry through the urinary tract. Dark blue indicates presence of the unitig, light blue absence. (EPS)

S3 Fig Correlation of odds ratio for all tested unitigs for the two main portal of entry. (EPS)

S4 Fig Structure of the *pap* operon island and relative position of the putative *opgE* gene. a) Position and relative orientation of the *pap* operon and the putative *opgE* gene is shown for one sample strain belonging to each major *E*. *coli* phylogroup. Genes colored in blue have at least one associated unitig mapped to it (using the entry through the urinary tract as target variable), grey otherwise. b) Distance between the putative *opgE* gene and the *pap* operon in those strains in which the two genetic elements are encoded in the same contig, and c) Distance between the *pap* operon and the edge of the contig in those strains in which the putative *opgE* gene is encoded in a different contig.
(EPS)

S5 Fig Genome-wide association analysis results on the combined dataset, including an association with B2 isolates only. a) Number of unitigs passing the multiple testing correction p-value threshold for each target phenotype. b) Number of genes with significantly associated unitigs mapped to them for each target phenotype. c) Manhattan plots for tested unitigs from B2 isolates mapping to *E*. *coli* IAI39 for each target phenotype; red dashed line indicates the p-value threshold used to call significant associations.
(EPS)

S1 Table Clinical variables for both cohorts, in its original form and after imputation of missing values. (XLSX)

S2 Table Lineage associations. (XLSX)

S3 Table Associated unitigs. (XLSX)

S4 Table Genes to which associated unitigs map to (see Materials and methods for mapping and filtering). (XLSX)

S1 Data Aminoacid sequence for each associated gene, sampled randomly for each gene cluster. (FAA)

## References

[1] Abernethy JK, Johnson AP, Guy R, Hinton N, Sheridan EA, Hope RJ. Thirty day all-cause mortality in patients with Escherichia coli bacteraemia in England. Clin Microbiol Infect. 2015;21: 251.e1–8. doi: 10.1016/j.cmi.2015.01.001 25698659

[2] Yoon E-J, Choi MH, Park YS, Lee HS, Kim D, Lee H, et al. Impact of host-pathogen-treatment tripartite components on early mortality of patients with Escherichia coli bloodstream infection: Prospective observational study. EBioMedicine. 2018;35: 76–86. doi: 10.1016/j.ebiom.2018.08.029 30139627 PMC6161478

[3] MacKinnon MC, McEwen SA, Pearl DL, Lyytikäinen O, Jacobsson G, Collignon P, et al. Increasing incidence and antimicrobial resistance in Escherichia coli bloodstream infections: a multinational population-based cohort study. Antimicrob Resist Infect Control. 2021;10: 131. doi: 10.1186/s13756-021-00999-4 34488891 PMC8422618

[4] Desjardins P, Picard B, Kaltenböck B, Elion J, Denamur E. Sex in Escherichia coli does not disrupt the clonal structure of the population: evidence from random amplified polymorphic DNA and restriction-fragment-length polymorphism. J Mol Evol. 1995;41: 440–448. doi: 10.1007/BF00160315 7563131

[5] Denamur E, Clermont O, Bonacorsi S, Gordon D. The population genetics of pathogenic Escherichia coli. Nat Rev Microbiol. 2021;19: 37–54. doi: 10.1038/s41579-020-0416-x 32826992

[6] Martínez JA, Soto S, Fabrega A, Almela M, Mensa J, Soriano A, et al. Relationship of phylogenetic background, biofilm production, and time to detection of growth in blood culture vials with clinical variables and prognosis associated with Escherichia coli bacteremia. J Clin Microbiol. 2006;44: 1468–1474. doi: 10.1128/JCM.44.4.1468-1474.2006 16597878 PMC1448679

[7] Jauréguy F, Carbonnelle E, Bonacorsi S, Clec’h C, Casassus P, Bingen E, et al. Host and bacterial determinants of initial severity and outcome of Escherichia coli sepsis. Clin Microbiol Infect. 2007;13: 854–862. doi: 10.1111/j.1469-0691.2007.01775.x 17617183

[8] Lefort A, Panhard X, Clermont O, Woerther P-L, Branger C, Mentré F, et al. Host factors and portal of entry outweigh bacterial determinants to predict the severity of Escherichia coli bacteremia. J Clin Microbiol. 2011;49: 777–783. doi: 10.1128/JCM.01902-10 21177892 PMC3067752

[9] Mora-Rillo M, Fernández-Romero N, Navarro-San Francisco C, Díez-Sebastián J, Romero-Gómez MP, Fernández FA, et al. Impact of virulence genes on sepsis severity and survival in Escherichia coli bacteremia. Virulence. 2015;6: 93–100. doi: 10.4161/21505594.2014.991234 25654604 PMC4603433

[10] de Lastours V, Laouénan C, Royer G, Carbonnelle E, Lepeule R, Esposito-Farèse M, et al. Mortality in Escherichia coli bloodstream infections: antibiotic resistance still does not make it. J Antimicrob Chemother. 2020;75: 2334–2343. doi: 10.1093/jac/dkaa161 32417924

[11] Falush D. Bacterial genomics: Microbial GWAS coming of age. Nature microbiology. 2016. p. 16059. doi: 10.1038/nmicrobiol.2016.59 27572652

[12] Mageiros L, Méric G, Bayliss SC, Pensar J, Pascoe B, Mourkas E, et al. Genome evolution and the emergence of pathogenicity in avian Escherichia coli. Nat Commun. 2021;12: 765. doi: 10.1038/s41467-021-20988-w 33536414 PMC7858641

[13] Biggel M, Xavier BB, Johnson JR, Nielsen KL, Frimodt-Møller N, Matheeussen V, et al. Horizontally acquired papGII-containing pathogenicity islands underlie the emergence of invasive uropathogenic Escherichia coli lineages. Nat Commun. 2020;11: 5968. doi: 10.1038/s41467-020-19714-9 33235212 PMC7686366

[14] Touchon M, Perrin A, de Sousa JAM, Vangchhia B, Burn S, O’Brien CL, et al. Phylogenetic background and habitat drive the genetic diversification of Escherichia coli. PLoS Genet. 2020;16: e1008866. doi: 10.1371/journal.pgen.1008866 32530914 PMC7314097

[15] Hendriks ACA, Reubsaet FAG, Kooistra-Smid AMDM, Rossen JWA, Dutilh BE, Zomer AL, et al. Genome-wide association studies of Shigella spp. and Enteroinvasive Escherichia coli isolates demonstrate an absence of genetic markers for prediction of disease severity. BMC Genomics. 2020;21: 138. doi: 10.1186/s12864-020-6555-7 32041522 PMC7011524

[16] Lees JA, Ferwerda B, Kremer PHC, Wheeler NE, Serón MV, Croucher NJ, et al. Joint sequencing of human and pathogen genomes reveals the genetics of pneumococcal meningitis. Nat Commun. 2019;10: 2176. doi: 10.1038/s41467-019-09976-3 31092817 PMC6520353

[17] Votintseva AA, Bradley P, Pankhurst L, Del Ojo Elias C, Loose M, Nilgiriwala K, et al. Same-Day Diagnostic and Surveillance Data for Tuberculosis via Whole-Genome Sequencing of Direct Respiratory Samples. J Clin Microbiol. 2017;55: 1285–1298. doi: 10.1128/JCM.02483-16 28275074 PMC5405248

[18] Chaguza C, Senghore M, Bojang E, Gladstone RA, Lo SW, Tientcheu P-E, et al. Within-host microevolution of Streptococcus pneumoniae is rapid and adaptive during natural colonisation. Nat Commun. 2020;11: 3442. doi: 10.1038/s41467-020-17327-w 32651390 PMC7351774

[19] Royer G, Darty MM, Clermont O, Condamine B, Laouenan C, Decousser J-W, et al. Phylogroup stability contrasts with high within sequence type complex dynamics of Escherichia coli bloodstream infection isolates over a 12-year period. Genome Med. 2021;13: 77. doi: 10.1186/s13073-021-00892-0 33952335 PMC8097792

[20] Galardini M, Clermont O, Baron A, Busby B, Dion S, Schubert S, et al. Major role of iron uptake systems in the intrinsic extra-intestinal virulence of the genus Escherichia revealed by a genome-wide association study. PLoS Genet. 2020;16: e1009065. doi: 10.1371/journal.pgen.1009065 33112851 PMC7592755

[21] Visscher PM, Hill WG, Wray NR. Heritability in the genomics era—concepts and misconceptions. Nat Rev Genet. 2008;9: 255–266. doi: 10.1038/nrg2322 18319743

[22] Jaillard M, Lima L, Tournoud M, Mahé P, van Belkum A, Lacroix V, et al. A fast and agnostic method for bacterial genome-wide association studies: Bridging the gap between k-mers and genetic events. PLoS Genet. 2018;14: e1007758. doi: 10.1371/journal.pgen.1007758 30419019 PMC6258240

[23] Lees JA, Mai TT, Galardini M, Wheeler NE, Horsfield ST, Parkhill J, et al. Improved Prediction of Bacterial Genotype-Phenotype Associations Using Interpretable Pangenome-Spanning Regressions. MBio. 2020;11. doi: 10.1128/mBio.01344-20 32636251 PMC7343994

[24] Earle SG, Wu CH, Charlesworth J, Stoesser N, Gordon NC, Walker TM, et al. Identifying lineage effects when controlling for population structure improves power in bacterial association studies. Nature Microbiology. 2016;1: 1–8. doi: 10.1038/nmicrobiol.2016.41 27572646 PMC5049680

[25] Ma KC, Mortimer TD, Duckett MA, Hicks AL, Wheeler NE, Sánchez-Busó L, et al. Increased power from conditional bacterial genome-wide association identifies macrolide resistance mutations in Neisseria gonorrhoeae. Nat Commun. 2020;11: 5374. doi: 10.1038/s41467-020-19250-6 33097713 PMC7584619

[26] Saber MM, Shapiro BJ. Benchmarking bacterial genome-wide association study methods using simulated genomes and phenotypes. Microb Genom. 2020;6. doi: 10.1099/mgen.0.000337 32100713 PMC7200059

[27] Strömberg N, Marklund BI, Lund B, Ilver D, Hamers A, Gaastra W, et al. Host-specificity of uropathogenic Escherichia coli depends on differences in binding specificity to Gal alpha 1-4Gal-containing isoreceptors. EMBO J. 1990;9: 2001–2010. 1693334 10.1002/j.1460-2075.1990.tb08328.x PMC551909

[28] Ebel W, Vaughn GJ, Peters HK 3rd, Trempy JE. Inactivation of mdoH leads to increased expression of colanic acid capsular polysaccharide in Escherichia coli. J Bacteriol. 1997;179: 6858–6861. doi: 10.1128/jb.179.21.6858-6861.1997 9352941 PMC179620

[29] Bhagwat AA, Jun W, Liu L, Kannan P, Dharne M, Pheh B, et al. Osmoregulated periplasmic glucans of Salmonella enterica serovar Typhimurium are required for optimal virulence in mice. Microbiology. 2009;155: 229–237. doi: 10.1099/mic.0.023747-0 19118363

[30] Bontemps-Gallo S, Madec E, Dondeyne J, Delrue B, Robbe-Masselot C, Vidal O, et al. Concentration of osmoregulated periplasmic glucans (OPGs) modulates the activation level of the RcsCD RcsB phosphorelay in the phytopathogen bacteriaDickeya dadantii. Environmental Microbiology. 2013. pp. 881–894. doi: 10.1111/1462-2920.12054 23253096

[31] Bontemps-Gallo S, Cogez V, Robbe-Masselot C, Quintard K, Dondeyne J, Madec E, et al. Biosynthesis of osmoregulated periplasmic glucans in Escherichia coli: the phosphoethanolamine transferase is encoded by opgE. Biomed Res Int. 2013;2013. Available: https://www.hindawi.com/journals/bmri/aip/371429/ doi: 10.1155/2013/371429 24228245 PMC3818809

[32] Landraud L, Jauréguy F, Frapy E, Guigon G, Gouriou S, Carbonnelle E, et al. Severity of Escherichia coli bacteraemia is independent of the intrinsic virulence of the strains assessed in a mouse model. Clin Microbiol Infect. 2013;19: 85–90. doi: 10.1111/j.1469-0691.2011.03750.x 22268649

[33] Picard B, Garcia JS, Gouriou S, Duriez P, Brahimi N, Bingen E, et al. The link between phylogeny and virulence in Escherichia coli extraintestinal infection. Infect Immun. 1999;67: 546–553. doi: 10.1128/IAI.67.2.546-553.1999 9916057 PMC96353

[34] Johnson JR, O’Bryan TT, Kuskowski M, Maslow JN. Ongoing Horizontal and Vertical Transmission of Virulence Genes and papA Alleles among Escherichia coli Blood Isolates from Patients with Diverse-Source Bacteremia. Infection and Immunity. 2001. pp. 5363–5374. doi: 10.1128/IAI.69.9.5363-5374.2001 11500406 PMC98646

[35] Jantausch BA, Hull SI. Restriction fragment length polymorphism of PCR amplifiedpapE gene products is correlated with complete serotype among uropathogenicEscherichia coliisolates. Microb Pathog. 1996;20: 351–360. doi: 10.1006/mpat.1996.0033 8831830

[36] Schubert S, Cuenca S, Fischer D, Heesemann J. High-pathogenicity island of Yersinia pestis in enterobacteriaceae isolated from blood cultures and urine samples: prevalence and functional expression. J Infect Dis. 2000;182: 1268–1271. doi: 10.1086/315831 10979932

[37] Schubert S, Picard B, Gouriou S, Heesemann J, Denamur E. Yersinia high-pathogenicity island contributes to virulence in Escherichia coli causing extraintestinal infections. Infect Immun. 2002;70: 5335–5337. doi: 10.1128/IAI.70.9.5335-5337.2002 12183596 PMC128248

[38] Bearden SW, Staggs TM, Perry RD. An ABC transporter system of Yersinia pestis allows utilization of chelated iron by Escherichia coli SAB11. J Bacteriol. 1998;180: 1135–1147. doi: 10.1128/JB.180.5.1135-1147.1998 9495751 PMC107000

[39] Mühldorfer I, Hacker J. Genetic aspects of Escherichia coli virulence. Microb Pathog. 1994;16: 171–181. doi: 10.1006/mpat.1994.1018 7522300

[40] Didelot X, Bowden R, Wilson DJ, Peto TEA, Crook DW. Transforming clinical microbiology with bacterial genome sequencing. Nat Rev Genet. 2012;13: 601–612. doi: 10.1038/nrg3226 22868263 PMC5049685

[41] Fricke WF, Rasko DA. Bacterial genome sequencing in the clinic: bioinformatic challenges and solutions. Nat Rev Genet. 2014;15: 49–55. doi: 10.1038/nrg3624 24281148

[42] Bertelli C, Greub G. Rapid bacterial genome sequencing: methods and applications in clinical microbiology. Clin Microbiol Infect. 2013;19: 803–813. doi: 10.1111/1469-0691.12217 23601179

[43] Bycroft C, Freeman C, Petkova D, Band G, Elliott LT, Sharp K, et al. The UK Biobank resource with deep phenotyping and genomic data. Nature. 2018;562: 203–209. doi: 10.1038/s41586-018-0579-z 30305743 PMC6786975

[44] Vöhringer HS, Sanderson T, Sinnott M, De Maio N, Nguyen T, Goater R, et al. Genomic reconstruction of the SARS-CoV-2 epidemic in England. Nature. 2021. doi: 10.1038/s41586-021-04069-y 34649268 PMC8674138

[45] Michaelsen TY, Bennedbæk M, Christiansen LE, Jørgensen MSF, Møller CH, Sørensen EA, et al. Introduction and transmission of SARS-CoV-2 B.1.1.7 in Denmark. bioRxiv. medRxiv; 2021. doi: 10.1101/2021.01.07.21249419 33442703 PMC7805463

[46] Van Hout CV, Tachmazidou I, Backman JD, Hoffman JX, Ye B, Pandey AK, et al. Whole exome sequencing and characterization of coding variation in 49,960 individuals in the UK Biobank. bioRxiv. 2019. p. 572347. doi: 10.1101/572347 PMC7759458 33087929

[47] Singer M, Deutschman CS, Seymour CW, Shankar-Hari M, Annane D, Bauer M, et al. The Third International Consensus Definitions for Sepsis and Septic Shock (Sepsis-3). JAMA. 2016;315: 801–810. doi: 10.1001/jama.2016.0287 26903338 PMC4968574

[48] Van Buuren S, Groothuis-Oudshoorn K. mice: Multivariate imputation by chained equations in R. J Stat Softw. 2011;45: 1–67.

[49] Venables WN, Ripley BD. Random and Mixed Effects. In: Venables WN, Ripley BD, editors. Modern Applied Statistics with S. New York, NY: Springer New York; 2002. pp. 271–300.

[50] Prjibelski A, Antipov D, Meleshko D, Lapidus A, Korobeynikov A. Using SPAdes De Novo Assembler. Curr Protoc Bioinformatics. 2020;70: e102. doi: 10.1002/cpbi.102 32559359

[51] Seemann T. Prokka: rapid prokaryotic genome annotation. Bioinformatics. 2014;30: 2068–2069. doi: 10.1093/bioinformatics/btu153 24642063

[52] Page AJ, Cummins CA, Hunt M, Wong VK, Reuter S, Holden MTG, et al. Roary: rapid large-scale prokaryote pan genome analysis. Bioinformatics. 2015;31: 3691–3693. doi: 10.1093/bioinformatics/btv421 26198102 PMC4817141

[53] Minh BQ, Schmidt HA, Chernomor O, Schrempf D, Woodhams MD, von Haeseler A, et al. IQ-TREE 2: New Models and Efficient Methods for Phylogenetic Inference in the Genomic Era. Mol Biol Evol. 2020;37: 1530–1534. doi: 10.1093/molbev/msaa015 32011700 PMC7182206

[54] Letunic I, Bork P. Interactive Tree Of Life (iTOL) v5: an online tool for phylogenetic tree display and annotation. Nucleic Acids Res. 2021;49: W293–W296. doi: 10.1093/nar/gkab301 33885785 PMC8265157

[55] Tonkin-Hill G, MacAlasdair N, Ruis C, Weimann A, Horesh G, Lees JA, et al. Producing polished prokaryotic pangenomes with the Panaroo pipeline. Genome Biol. 2020;21: 180. doi: 10.1186/s13059-020-02090-4 32698896 PMC7376924

[56] Lippert C, Casale FP, Rakitsch B, Stegle O. LIMIX: genetic analysis of multiple traits. bioRxiv. 2014. p. 003905. doi: 10.1101/003905

[57] Schweiger R, Kaufman S, Laaksonen R, Kleber ME, März W, Eskin E, et al. Fast and Accurate Construction of Confidence Intervals for Heritability. Am J Hum Genet. 2016;98: 1181–1192. doi: 10.1016/j.ajhg.2016.04.016 27259052 PMC4908190

[58] Ondov BD, Treangen TJ, Melsted P, Mallonee AB, Bergman NH, Koren S, et al. Mash: fast genome and metagenome distance estimation using MinHash. Genome Biol. 2016;17: 132. doi: 10.1186/s13059-016-0997-x 27323842 PMC4915045

[59] Lees JA, Galardini M, Bentley SD, Weiser JN, Corander J. pyseer: a comprehensive tool for microbial pangenome-wide association studies. Bioinformatics. 2018;34: 4310–4312. doi: 10.1093/bioinformatics/bty539 30535304 PMC6289128

[60] Lippert C, Listgarten J, Liu Y, Kadie CM, Davidson RI, Heckerman D. FaST linear mixed models for genome-wide association studies. Nature Methods. 2011. pp. 833–835. doi: 10.1038/nmeth.1681 21892150

[61] Li H. Aligning sequence reads, clone sequences and assembly contigs with BWA-MEM. arXiv [q-bio.GN]. 2013. Available: http://arxiv.org/abs/1303.3997

[62] Quinlan AR, Hall IM. BEDTools: a flexible suite of utilities for comparing genomic features. Bioinformatics. 2010;26: 841–842. doi: 10.1093/bioinformatics/btq033 20110278 PMC2832824

[63] Dale RK, Pedersen BS, Quinlan AR. Pybedtools: a flexible Python library for manipulating genomic datasets and annotations. Bioinformatics. 2011;27: 3423–3424. doi: 10.1093/bioinformatics/btr539 21949271 PMC3232365

[64] Huerta-Cepas J, Forslund K, Coelho LP, Szklarczyk D, Jensen LJ, von Mering C, et al. Fast Genome-Wide Functional Annotation through Orthology Assignment by eggNOG-Mapper. Mol Biol Evol. 2017;34: 2115–2122. doi: 10.1093/molbev/msx148 28460117 PMC5850834

[65] Cingolani P, Platts A, Wang LL, Coon M, Nguyen T, Wang L, et al. A program for annotating and predicting the effects of single nucleotide polymorphisms, SnpEff: SNPs in the genome of Drosophila melanogaster strain w1118; iso-2; iso-3. Fly. 2012;6: 80–92. doi: 10.4161/fly.19695 22728672 PMC3679285

[66] Danecek P, Bonfield JK, Liddle J, Marshall J, Ohan V, Pollard MO, et al. Twelve years of SAMtools and BCFtools. Gigascience. 2021;10. doi: 10.1093/gigascience/giab008 33590861 PMC7931819

[67] Vaser R, Adusumalli S, Leng SN, Sikic M, Ng PC. SIFT missense predictions for genomes. Nat Protoc. 2016;11: 1–9. doi: 10.1038/nprot.2015.123 26633127

[68] UniProt: the universal protein knowledgebase in 2021. Nucleic Acids Res. 2021;49: D480–D489. doi: 10.1093/nar/gkaa1100 33237286 PMC7778908

[69] McKinney W. Data Structures for Statistical Computing in Python. Proceedings of the 9th Python in Science Conference. SciPy; 2010. doi: 10.25080/majora-92bf1922-00a

[70] Harris CR, Millman KJ, van der Walt SJ, Gommers R, Virtanen P, Cournapeau D, et al. Array programming with NumPy. Nature. 2020;585: 357–362. doi: 10.1038/s41586-020-2649-2 32939066 PMC7759461

[71] Virtanen P, Gommers R, Oliphant TE, Haberland M, Reddy T, Cournapeau D, et al. SciPy 1.0: fundamental algorithms for scientific computing in Python. Nat Methods. 2020;17: 261–272. doi: 10.1038/s41592-019-0686-2 32015543 PMC7056644

[72] Hunter JD. Matplotlib: A 2D Graphics Environment. Computing in Science Engineering. 2007;9: 90–95.

[73] Waskom M. seaborn: statistical data visualization. J Open Source Softw. 2021;6: 3021.

[74] Cock PJA, Antao T, Chang JT, Chapman BA, Cox CJ, Dalke A, et al. Biopython: freely available Python tools for computational molecular biology and bioinformatics. Bioinformatics. 2009;25: 1422–1423. doi: 10.1093/bioinformatics/btp163 19304878 PMC2682512

[75] Pritchard L, White JA, Birch PRJ, Toth IK. GenomeDiagram: a python package for the visualization of large-scale genomic data. Bioinformatics. 2006;22: 616–617. doi: 10.1093/bioinformatics/btk021 16377612

[76] Kluyver T, Ragan-Kelley B, Pérez F, Granger BE, Bussonnier M, Frederic J, et al. Jupyter Notebooks-a publishing format for reproducible computational workflows. ELPUB. 2016. pp. 87–90.

[77] Mölder F, Jablonski KP, Letcher B, Hall MB, Tomkins-Tinch CH, Sochat V, et al. Sustainable data analysis with Snakemake. F1000Res. 2021;10. doi: 10.12688/f1000research.29032.2 34035898 PMC8114187

[78] Analytics C. Anaconda software distribution. computer software. vers. 2–2.4. 0. 2015.

[79] Grüning B, Dale R, Sjödin A, Chapman BA, Rowe J, Tomkins-Tinch CH, et al. Bioconda: sustainable and comprehensive software distribution for the life sciences. Nat Methods. 2018;15: 475–476. doi: 10.1038/s41592-018-0046-7 29967506 PMC11070151

